# Impact of Season and OvSynch + GnRH on Day 5 After Artificial Insemination (AI) on the Heat Detection and Conception Rates of Cooled High-Yielding Holstein Cows

**DOI:** 10.3390/ani15060777

**Published:** 2025-03-09

**Authors:** Silviu-Ionuț Borş, Vasile Vintilă, Alina Borş, Viorel-Cezar Floriștean

**Affiliations:** 1 Research and Development Station for Cattle Breeding Dancu, 707252 Iasi, Romania; vasilevintilais@gmail.com; 2 “Ion Ionescu de la Brad” Iasi University of Life Sciences, 700490 Iasi, Romania; alina.bors@iuls.ro (A.B.); viorel.floristean@iuls.ro (V.-C.F.)

**Keywords:** cooling, high-yielding cows, heat stress, conception rate

## Abstract

If global warming persists, new strategies will be necessary to keep high-yielding dairy cows’ reproduction at a thermo-neutral level. In this study, two years of data from around 2000 cooled cows from a dairy farm in northeastern Romania were analyzed. In both years, an automated cooling system was used to maintain the temperature–humidity index (THI) within the range of 65–75 when environmental conditions exceeded the upper limit of this range. The univariate analysis shows that there may be a potential association between parity and the heat detection rate (*p* < 0.25). Additionally, the analysis suggests possible associations between conception rate and season, parity, and 4 + AIs (*p* < 0.25). These findings warrant further investigation through multivariate analysis. Summer season had the biggest impact (*p* = 0.001) on poor conception rate, according to the multivariate logistic regression analysis. Notably, the effects on conception rates extended into autumn (*p* = 0.004) in cooled high-yielding dairy cows. As expected, repeat breeding (4 + AIs) and parity (3 + lact.) impacted reproduction (*p* = 0.05). However, there was no association between OvSynch and OvSynch + GnRH on day 5 after AI and conception rate (*p* > 0.05). According to data from the current study, we concluded that the current cooling systems need to be improved to maintain reproduction at a thermo-neutral level for high-yielding dairy cows.

## Simple Summary

In heat-stressed, high-yielding dairy cows, the current methods for managing reproduction still need improvement. Future advancements will require new strategies to minimize additional interventions and maintain reproduction at a thermo-neutral level in high-yielding dairy cows. As a result, the development of new strategies in the dairy cow industry poses a significant challenge for improving reproductive performances. Our study suggests that high-yielding dairy cows raised in a temperature-controlled climate still experienced a lower conception rate during the summer season. This effect can last into autumn, even when a standard cooling system is used. Additionally, the OvSynch + GnRH on day 5 after artificial insemination (AI) produced results similar to those of the OvSynch protocol.

## 1. Introduction

Climate change is significantly impacting cattle reproduction and management worldwide, particularly affecting high-yielding dairy cows. Additionally, several factors are driving this transformation, including the rapid advancement of technologies designed to enhance dairy cow reproductive management, removing quotas in Europe, and the substantial growth in herd and farm sizes [1]. Recent estimates of annual financial losses from heat stress primarily stem from seasonal infertility, reduced milk quality and yield, and increased veterinary costs [2,3,4]. 

The reproductive performance of dairy cattle is negatively affected during the hot season due to both direct and indirect impacts on their reproductive systems [2]. Until the early 2000s, dairy genetic selection programs in dairy-producing countries primarily focused on increasing the milk yield, often neglecting other important dairy traits such as fertility and health [5,6].

Seasonal patterns of estrus detection, time to first service, and conception rates in dairy cows show consistently lower rates during summer than in winter [7]. Additionally, the effects of heat stress on fertility seem to extend into the autumn [8,9,10]. The low fertility typical of the warm months (June to September) persists into autumn (October and November), even though the cows are no longer exposed to heat stress [11].

For many years, researchers have been searching for a solution to mitigate the harmful effects of heat stress. The tested strategy has yielded varying degrees of success, but no single strategy has successfully restored its reproductive or productive performance to thermo-neutral levels [12]. There are no published studies that examine how seasons and the modified OvSynch protocol with GnRH 5 days post-AI impact heat detection and conception rates, particularly regarding summer infertility in high-yielding dairy cows raised in climate-controlled environments. Therefore, it remains unclear whether the reproduction of dairy cows is affected during the summer and autumn seasons on cooled dairy farms. Thus, this study evaluates how season, parity, and the modified OvSynch protocol with GnRH 5 days post-AI affect heat detection and conception rates in cooled high-yielding dairy cows raised in a temperate continental climate.

## 2. Materials and Methods

### 2.1. Ethical Approval

All the dairy cows were handled in accordance with the directives of the European Union on the protection of animals used for scientific purposes (Dir 2010/63/EU). The study protocol was approved by the Ethics Committee of Faculty of Veterinary Medicine, University of Life Sciences, 700489 Iasi, Romania. Efforts were made to minimize animal handling and stress.

### 2.2. Cattle Herd Management and Study Design

#### 2.2.1. Study Animals and Management

A northeastern Romanian Holstein-Friesian dairy herd (n = 2000) served as the subject of this study. The average number of lactating cows in the herd was around 800 during the study period, and the average milk output per cow was 14,030 kg/305 days. The number of nulliparous cows was roughly 865 in 2022 and 1043 in 2023, with a 14-month waiting period.

The cows were housed in free-stall barns with concrete floors and straw beds and fed a Total Mixed Ration twice per day with ad libitum water access, according to the level of milk production and cow size. To keep the animals healthy, standard management practices, including a cooling system coupled with a weather station (CMPimpianti, Calvisano (BS)—Italy) for hot conditions, were followed. Each barn is equipped with a THI controller, which is also present in the milking area. This system is linked to a central unit that manages the fans and water sprayers. The temperature–humidity index (THI), was automatically computed using the following Kelly and Bond (1971) [13] formula: THI = (1.8 × AT + 32) − [(0.55 − 0.0055 × RH) × (1.8 × AT − 26)]. The coolers turn on automatically when the THI reaches 65, operating at low speeds. The speeds increase as THI values rise, reaching a maximum of 75. Water sprayers are activated when the THI exceeds 75. They are only used on extremely hot summer days and operate for a short duration. However, during the summer season, the THI usually ranges between 70 and 75. During the spring and autumn months, the water sprayers are not in operation since the THI values typically remain below this threshold. The farm milked about 800 cows three times a day at 0400 h, 1200 h, and 1900 h, for a daily average of 46 kg milk/cow/day.

Estrous cows were identified using the AfiMilk (AfiMilk, Kibbutz Afikim, Israel) daily estrus report, and an experienced veterinarian team examined each one. Signs such as attempting to mount other cows, chasing herd mates, restlessness, chin resting, sniffing herd mates’ vaginas, bellowing, congestion, relaxation, and mucus discharge from the vulva were considered suggestive of estrus. Additionally, the visualization of a cow standing while being mounted was regarded as a definitive sign of estrus. AfiMilk recorded the cows that did not exhibit any signs of estrous, and these cows were added to the anestrous group.

The veterinary team performed the ultrasound examinations and hormone injections. A 5–7.5 MHz rectal convex probe (BCF EasyScan, BCF Ultrasound Australasia, Mitcham, VIC, Australia) was used to scan the uterus and diagnose pregnancy. Pregnancy confirmation was based on visualizing an anechoic fluid-filled uterine horn and the presence of an embryo, along with a corpus luteum (CL) on the same side of the uterus. The first pregnancy diagnosis was performed using transrectal ultrasonography on day 30 after AI and confirmed on day 60. The non-pregnant cows on day 30 after AI were included in the day’s open group and underwent the OvSynch protocol as anestrus cows. The data obtained from the first pregnancy diagnosis were utilized by the AfiMilk management software (AfiMilk, Kibbutz Afikim, Israel) to assess conception rates.

#### 2.2.2. Study Groups

During the study period from January 2022 to December 2023, the cows were grouped after calving based on days in milk (DIM), milk production, and parity. The calving dates, breeding dates, and DIM were obtained from the AfiMilk management software (AfiMilk, Kibbutz Afikim, Israel). The voluntary waiting period was set at 90 days during the studied period. After the end of the voluntary waiting period, the cows underwent AI at natural estrus, and the anestrous cows were subjected to the OvSynch protocol. The OvSynch protocol consists in administering the GnRH (dephereline: gonadorelin acetate [6-DPhe]; Gonavet Veyx, Veyx-Pharma GmbH, Schwarzenborn, Germany) on day 0, PGF2α (Cloprostenol, PGF Veyx, Veyx-Pharma GmbH, Schwarzenborn, Germany) on day 7 and 8, GnRH on day 9, and AI after 12 h by the GnRH treatment. From January 2022 to December 2022, approximately all the cows, starting with the first AI, were included in the G0 group, which received the above reproductive program. For the period from January 2023 to December 2023, each cow in the herd, starting with the first AI, was included in the G5 group and received the same reproductive program as in 2022 but, in addition, a single dose of GnRH (dephereline: gonadorelin acetate [6-DPhe]; Gonavet Veyx, Veyx-Pharma GmbH, Schwarzenborn, Germany) on day 5 after AI to induce ovulation and aCL formation. About 75% of AIs occurred during natural estrus in both years, with the remaining 25% occurring during an OvSynch protocol or OvSynch protocol + GnRH 5 days post-AI.

### 2.3. Statistical Analysis

For statistical analyses, cow parity was classified as either nulliparous, primiparous, or multiparous. The groups were categorized based on parity, season, G0 and G5 classification, and the number of AIs. The calving season was classified into four categories: spring (March to May), summer (June to August), autumn (September to November), and winter (December to February). Spring was chosen as the reference season due to its favorable weather conditions. Statistical analyses were conducted using the SAS program (ver. 9.4; SAS Institute, Cary, NC, USA). The odds ratio (OR) and 95% confidence interval (CI) were calculated during the logistic regression. The results are presented as proportions and ORs with their respective 95% CIs. A *t*-test was used to identify significant main effects. A *p*-value of <0.05 was considered significant. The analysis of risk factors potentially associated with heat detection and conception rates was conducted in two steps using logistic regression. In the first step, we performed a univariate analysis of each hypothesized risk factor (independent variable) and selected those with a *p*-value of less than 0.25 for further multivariable logistic regression analysis. We assessed the model’s fitness using the Hosmer and Lemeshow goodness-of-fit test. The results were presented as *p*-values and odds ratios (ORs) with 95% confidence intervals (95% CIs). 

## 3. Results

During the period under consideration, the studied high-yielding dairy farm presented a heat detection rate ranging from 65.1% in summer to 72.9% in winter, with no significant difference in the G0 group (Table 1), while it was higher (*p* < 0.05) in summer (60.8%) than in spring (67.2%) in the G5 group (*p* < 0.05, Table 2).

The cumulative conception rates ranged from 42.2% in the G0 group to 40.7% in the G5 group. The probability of conception following AI was influenced by the cow’s parity, season, and incidence of repeat breeding. In both groups, the likelihood of conception was lower (*p* < 0.05) during the summer (36.8% in the G0 group and 30.2% in the G5 group) and autumn seasons (40.7% in the G0 group and 39.8% in the G5 group) than in the spring (47.9% in the G0 group and 48.3 in the G5 group) (Table 1 and Table 2). Additionally, in the G0 group, nulliparous and primiparous cows exhibited a higher conception rate (54.7% and 49.6%) than multiparous cows (40.2% and 37.1%, *p* < 0.05). In the same way, the G5 group presented higher conception rates (*p* < 0.05) in nulliparous (58.7%) and primiparous cows (45.8%) than in multiparous ones (36.5% and 37.6%). In both groups, the first three AIs presented higher conception rates (ranged between 46.4–52.4% in the G0 group and 44.1–52.3% in the G5 group), while 4 + AIs affected the likelihood of conception (*p* < 0.05), and the values were similar (34.7% in the G0 group and 38.1% in the G5 group; Table 1 and Table 2).

The associations (univariate analysis) between the independent variables: season, parity, years, and heat detection rate are presented in Table 3. There was a potential association between the heat detection rate and the parity (*p* < 0.25); the multiparous cows exhibited a lower heat detection rate. 

The associations (univariate analysis) between the independent variables: season, parity, number of AI, OvSynch, and OvSynch + GnRH on day 5 after AI and the conception rate are presented in Table 4. Regarding the risk factor analysis, there is a potential association between the season (summer and autumn), parity (multiparous), 4 + AIs, and conception rate (*p* < 0.25). 

Using a multivariate logistic regression analysis for the estrus detection rate and conception rate of high-yielding Holstein cows (Table 5), we observed an association between summer and autumn seasons, parity (3 + lact.), repeat breeding (4 + AIs), and poor conception rate (*p* < 0.05). The summer season was found to have a significant impact (*p* = 0.001) on the lower conception rate in cooled high-yielding dairy cows, followed by the autumn season (*p* = 0.004), 3 + lactations, and 4 + AIs (*p* = 0.05). No association was observed between the independent variable and heat detection rate (*p* > 0.05) in multivariate logistic regression analysis. 

## 4. Discussion

Efforts were made to study the effects of independent factors on heat detection and conception rates in cooled high-yielding dairy cows. This study reveals that, for cooled high-yielding dairy cows, the summer season had the most significant impact on conception rates, followed by the autumn season. This finding is particularly interesting because the atmospheric THI decreases in autumn compared to summer. High submission rates for AI are essential for achieving a 365-day calving interval in seasonal calving herds. Therefore, an effective and practical method for identifying each cow in estrus is necessary [1]. The primary behavioral sign indicating an estrous period is standing to be mounted, which helps determine the optimal time for insemination [14]. However, many veterinarians recognize that visually detecting estrus in dairy cows during the summer can be challenging [15]. Thus, poor estrus detection affects dairy cows’ reproduction during the hot season [7]. Surprisingly, in this study, the estrus detection rates were higher in both years with only minor variations, particularly during the summer of 2023. According to the activity monitoring system, 95% of the cows that were identified to be in estrus ovulated, whereas only 5% did not ovulate within seven days of the induction of luteolysis [16]. Heat detection rates, or submission rates, differ among herds, with 30% to 70% of cows showing estrous behavior being detected in estrus [1]. In high-yielding Holstein cows, the percentage of cows that exhibit standing heat, where they allow themselves to be mounted by other cows, has decreased. This reduction makes it more challenging to detect estrus [17]. A study conducted by Roelofs et al. (2005) [17] found that only 58% of cows were observed in standing estrus. This finding aligns with the heat detection rate recorded during the summer in this study (65.1% in 2022 and 60.8% in 2023); however, it differs from the higher heat detection rates observed in the other three seasons. One possible explanation for these differences is that, on the studied farm, the cooling system activates the coolers at a THI of 65. This activation occurs not only during the summer season, and this enhances the comfort of the cows in all seasons, leading to an increase in their estrus behaviors. 

However, during the summer season, which is the hottest period in Romania, both groups experienced lower conception rates despite the intensive use of standard cooling devices on the farm. We also noticed that conception rates declined in the autumn. This association was confirmed through a multivariate logistic regression analysis, in which the summer season emerged as the greatest risk factor impacting high-yielding cow reproduction, followed by the autumn season. Possibly, the cooling system cannot supply the cows’ needs taking into account the high milk production (>14,000 kg milk/305 days), or the mechanism that affects reproduction in this period is unknown. Intensive cooling, which requires ten cooling periods, is essential to prevent a decline in milk production among high-yielding cows (those producing over 13,000 kg/year) [18]. A study comparing five and eight cooling periods, each lasting 45 min, found that eight cooling periods led to better physiological and behavioral responses. These improvements were indicated by lower rectal temperatures, reduced respiratory rates, and increased time spent lying down and ruminating [19]. Furthermore, the eight cooling periods increased dry matter intake (27.0 kg/day compared to 24.7 kg/day) and improved milk production (40.1 kg/day versus 36.6 kg/day). The dairy farm studied continuously operated the coolers after the THI reached 65 and kept the THI around 75 most of the time during the summer season.

Low reproductive performance in the summer and autumn seasons can be also associated with the different thermo-neutral levels of high-yielding dairy cows. High-yielding dairy cows generate more metabolic heat compared to dry cows. For example, cows producing 18.5 kg of milk per day and those producing 31.6 kg per day have been found to produce 27.3% and 48.5% more metabolic heat (measured in kJ/kgW^0.75^ per hour), respectively, compared to dry cows [20]. While we can reduce heat stress impact on reproduction, complete elimination is not possible. Thus, data from the Israeli dairy herd indicate that, with efficient cooling management, the conception rate during the summer is 14.9% lower compared to that in winter (27.7% in summer versus 42.6% in winter) [21]. Further research is crucial to evaluate the effects of heat stress on reproduction in high-yielding dairy cows and to create innovative cooling methods for dairy farms.

Another possible explanation for low conception rates during autumn may be due to how prolonged heat stress affects fertilization, rather than the reproductive management practices on the dairy farm. It has been suggested that heat stress during the hot months could have a lasting effect on the antral follicles, which will develop into large dominant follicle, 40–50 days later [22,23]. The effects of heat stress extend beyond the hot months, impacting the subsequent cooler months (autumn) and leading to long-term consequences throughout the year [24,25]. It takes a period of two to five estrous cycles (40 to 105 days) for recovery from summer heat stress and for competent oocytes to appear in the following autumn [22,26]. This prolonged impact of heat stress on the oocytes may explain the reduced conception observed in autumn when high-yielding cows are no longer exposed to environmental thermal stress.

An alternative method to enhance fertility in heat-stressed dairy cows is the use of hormones to stimulate reproduction. The study conducted by Schmitt et al. (1996) [27] investigated the induction of an accessory corpus luteum (CL) using a GnRH agonist (Buserelin, 8 mg) or hCG (3000 IU). The aim was to increase plasma progesterone levels and enhance conception rates in nulliparous and lactating cows. The results showed that both hormones were effective in inducing the formation of an accessory corpus luteum and subsequently increasing progesterone concentrations. However, the study found that these treatments did not improve conception rates in either nulliparous or dairy cows during the summer. Several studies have shown that administering GnRH between days 5 and 15 post-AI can increase conception rates during the warm season by approximately 15 percentage points [28,29]. In these studies, the average milk production was around 11,000 kg/305 days, which was lower than that of the farm being investigated (14,030 kg/305 days). This treatment may not enhance conception rates on high-yield dairy farms, particularly those that need an extra cooling system during extremely hot periods. Several risk factors can contribute to low conception rates in high-yielding dairy cows. These risk factors include age, parity, body condition, milk yield, environmental conditions, as well as imbalances during the peri- and postpartum periods [30]. In the current study, the identified risk factors for low conception rate were season (summer and autumn), parity (3 + lactations), and 4 + AIs. 

Our previous study [31] demonstrated that administering the GnRH 7–14 days after AI improved reproductive activity in repeat breeder cows. This finding indicates that administering gonadorelin later in the luteal phase may increase the survival of embryos in repeat breeder dairy cows or infertile cows [31]. According to the results of Doležel et al. (2017) [32], inducing aCL formation through gonadorelin administration was found to be more efficient in cows treated on day 5 following AI, as compared to cows treated on days 6 or 7. Yan et al. (2016) [33] discovered that administering progesterone to dairy cows between 3 and 7 days after AI yielded benefits, while supplementation given earlier or later did not. Other studies have discovered that treatment with GnRH 10 days after AI is also conducive to pregnancy after AI [34]. In this regard, the farm owner administered the GnRH agonist on day 5 after AI in 2023, but reproduction did not improve in any season, as the reproductive parameters remained approximately the same compared with the previous year in which this strategy was not used. In addition, the current study shows that the conception rate in cows subjected to the OvSynch protocol, which received GnRH on day 5 after AI, was similar to that in the cows subjected to the OvSynch protocol. Similar findings have been observed with exogenous progesterone administered through the CIDR intravaginal delivery device [35]. 

In this study, the conception rates were lower in multiparous cows than in nulliparous and primiparous high-yielding dairy ones. This aligns with the findings of Fodor et al. (2019) [36], who reported a significantly better conception rate in primiparous cows compared to multiparous cows. This difference may be related to the larger uterine size in multiparous cows than in nulliparous and primiparous cows as was revealed by the study of Koyama et al. (2023) [37]. Although the interval from estrus to ovulation is similar among nulliparous, primiparous, and multiparous cows, the optimal timing for insemination in multiparous cows is more restricted than in nulliparous and primiparous cows. Therefore, to improve the conception rate in multiparous cows, it is crucial to identify the optimal time for AI [37]. 

The conception rates of high-yielding dairy cows vary with the number of AIs. As expected, the cows with four or more AIs presented a low conception rate compared with that for the first three AIs in both years. Hormonal alterations are significant factors contributing to subfertility in repeat breeder cows. Key issues include late postovulatory rise in plasma progesterone levels, abnormal follicular dynamics, delayed ovulation, and reduced oocyte quality [38]. In addition, the subfertility of repeat breeder cows may be linked to low progesterone concentrations during the early stages of embryo development or poor oocyte quality in the late luteal phase [39]. 

## 5. Study Limitations

This study had limitations including the lack of a control group (non-cooling cows raised in the same condition). Some limitations were inevitable due to this study’s design constraints [39]. The cows’ owner accepted this study due to the field conditions, without supplementary evaluation of the cows (ultrasound evaluation, blood collection, creating a control group), which would create additional stress for the animals. Despite these limitations, data from the current study could be useful for high-yielding dairy farms or veterinarians. Inducing heat stress experimentally in such a large herd poses significant challenges. First, isolating a group of animals under different environmental conditions is not feasible within the current farm infrastructure. Additionally, constant monitoring and periodic adjustments of the conditions would contradict the automatic nature of the cooling system in place. Furthermore, this approach could have long-term consequences on the health of the control group, ultimately influencing the results of this study. On a high-yielding dairy farm, animal housing is optimized for productivity, making it difficult to create a non-cooled section without disrupting overall farm operations. Researchers are now exploring questions that may not be appropriate for a randomized controlled trial due to practical or ethical concerns. Nostalgia for the “magic” of large randomized controlled trials and the ongoing debate about “randomized controlled trials vs. real-world evidence” are unhelpful, both for assessing safety and effectiveness [40,41].

## 6. Conclusions

High-yielding dairy cows can be affected by climate variations even when a standard cooling system is used. While the heat detection rate remains unaffected by the season, the conception rate is affected by the hot months from the summer. This negative impact can persist into the autumn for cooled high-yielding dairy cows. Additionally other risk factors, like parity (3 + lactations) and 4 + AIs can adversely affect reproduction. Administering GnRH on day 5 after AI in natural estrus or OvSynch protocol did not improve reproduction in high-yielding dairy cows. Thus, in the context of climate change, innovative cooling systems are needed to maintain reproduction at thermo-neutral levels in high-yielding dairy cows. 

## Figures and Tables

**Table 1:** Odds ratio (OR) of variables included in the logistic regression model of the probability of heat detection/conception in the G0 group.

Variable Level	Reproductive Parameter	OR	95% CI	*p*-Value
**Season**	**Heat detection rate, %** **(number of cows)**			
Spring	68.5 (417/609)	Reference		
Summer	65.1 (413/634)	0.86	0.679–1.089	>0.05
Autumn	71.8 (587/817)	1.17	0.934–1.477	>0.05
Winter	72.9 (468/642)	1.24	0.970–1.581	>0.05
**Season**	**Conception rate, %** **(number of cows)**			
Spring	47.9 (200/417)	Reference		
Summer	36.8 (152/413)	0.62	0.477–0.839	<0.05
Autumn	40.7 (239/587)	0.73	0.574–0.965	<0.05
Winter	43.8 (205/468)	0.82	0.644–1.107	>0.05
**Cow parity**	**Conception rate, %** **(number of cows)**			
Nulliparous	54.7 (473/865)	Reference		
1st lact.	49.6 (373/752)	0.82	0.671–0.992	>0.05
2nd lact.	40.2 (199/495)	0.56	0.445–0.697	<0.05
3 + lact.	37.1 (246/664)	0.49	0.404–0.611	<0.05
**No. of AIs**	**Conception rate, %** **(number of cows)**			
1 AI	52.4 (724/1381)	Reference		
2 AIs	46.7(319/684)	0.79	0.660–0.952	>0.05
3 AIs	46.4 (172/371)	0.78	0.623–0.986	>0.05
4 + AIs	34.7 (118/340)	0.48	0.377–0.617	<0.05

OR = odds ratio, CI = confidence interval.

**Table 2:** Odds ratio (OR) of variables included in the logistic regression model of the probability of heat detection/pregnancy/conception in the G5 group.

Variable Level	Reproductive Parameter	OR	95% CI	*p*-Value
**Season**	**Heat detection rate, %** **(number of cows)**			
Spring	67.2 (518/771)	Reference		
Summer	60.8 (513/844)	0.76	0.617–0.928	<0.05
Autumn	71.5 (590/825)	1.23	0.991–1.518	>0.05
Winter	69.5 (533/767)	1.11	0.897–1.379	>0.05
**Season**	**Conception rate, %** **(number of cows)**			
Spring	48.3 (250/518)	Reference		
Summer	30.2 (155/513)	0.46	0.354–0.610	<0.05
Autumn	39.8 (235/590)	0.7	0.543–0.904	<0.05
Winter	44.5 (237/533)	0.88	0.661–1.111	>0.05
**Cow parity**	**Conception rate, %** **(number of cows)**			
Nulliparous	58.7 (612/1043)	Reference		
1st lact.	45.8 (207/452)	0.59	0.743–0.476	>0.05
2nd lact.	36.5 (262/717)	0.41	0.333–0.493	<0.05
3 + lact.	37.6 (264/703)	0.6	0.348–0.515	<0.05
**No. of AIs**	**Conception rate, %** **(number of cows)**			
1 AI	52.3 (760/1452)	Reference		
2 AIs	44.1 (311/706)	0.72	0.598–0.858	>0.05
3 AIs	45.6 (181/397)	0.76	0.610–0.953	>0.05
4 + AIs	38.1 (137/360)	0.55	0.442–0.709	<0.05

OR = odds ratio, CI = confidence interval.

**Table 3:** Univariate analysis of risk factors associated with poor heat detection rate in cooled high-yielding dairy cows.

Variable	OR	95% CI	*p*-Value
**Season (n = 5691)**			
Spring	1		
Summer	0.99	0.86–1.15	NS
Autumn	0.83	0.71–0.97	NS
Winter	0.86	0.73–1.01	NS
**Parity (n = 5691)**			
Nulliparous	2.1		
1st lact.	1.25	1.07–1.46	NS
2nd lact.	0.87	0.75–0.99	*p* < 0.25
3 + lact.	0.86	0.73–1.01	*p* < 0.25
**Year (n = 5691)**			
2022	1.31		
2023	1.13	1.01–1.26	NS

OR = odds ratio, CI = confidence interval, NS = *p* > 0.25.

**Table 4:** Univariate analysis of risk factors associated with poor conception rate in cooled high-yielding dairy cows.

Variable	OR	95% CI	*p*-Value
**Season (n = 5691)**			
Spring	0.93		
Summer	1.86	1.53–2.26	*p* < 0.25
Autumn	1.37	1.15–1.65	*p* < 0.25
Winter	1.17	0.97–1.42	NS
**Parity (n = 5691)**			
Nulliparous	1.32		
1st lact.	1.42	1.23–1.64	NS
2nd lact.	2.15	1.85–2.49	*p* < 0.25
3 + lact.	2.22	1.92–2.55	*p* < 0.25
**No. of AIs (n = 5691)**			
1 AI	1.10		
2 AIs	1.33	1.17–1.51	NS
3 AIs	1.29	1.1–1.52	NS
4 + AIs	3.02	2.57–3.55	*p* < 0.25
**Protocol (n = 1423)**			
OvSynch	1.41		
OvSynch + GnRH 5d Post-AI	1.20	1.07–1.35	NS

OR = odds ratio, CI = confidence interval, NS = *p* > 0.25.

**Table 5:** Multivariate logistic regression analysis of risk factors associated with poor heat detection and conception rates of cooled high-yielding Holstein cows.

Variable	Heat Detection Rate		
	OR	95% CI	*p*-Value
2nd lact.	1.06	0.8–1.41	0.650
3 + lact.	0.83	0.48–1.41	0.489
		**Conception rate**	
Summer	0.97	0.96–0.99	0.001
Autumn	0.65	0.50–085	0.004
2nd lact.	1.36	0.98–1.80	0.061
3 + lact.	1.01	0.75–1.30	0.05
4 + AIs	1.08	0.88–1.29	0.05

OR = odds ratio, CI = confidence interval.

## Data Availability

The data that support the findings of this study are available upon reasonable request.
